# Prognostic Value of Regadenoson Stress Perfusion CMR

**DOI:** 10.3390/medsci14020190

**Published:** 2026-04-10

**Authors:** Javier Muñiz-Sáenz-Diez, Ana Ezponda, Meylin Caballeros, Ana de la Fuente, Nahikari Salterain, Gorka Bastarrika

**Affiliations:** 1 Department of Cardiology, Clínica Universidad de Navarra, 31008 Pamplona, Spain; munhiz@gmail.com (J.M.-S.-D.); nsalterain@unav.es (N.S.); 2 Department of Radiology, Clínica Universidad de Navarra, 31008 Pamplona, Spain; aezponda@unav.es; 3 Department of Radiology, Clínica Universidad de Navarra, 28027 Madrid, Spain; fcaballeros@unav.es; 4 Department of Cardiology, Clínica Universidad de Navarra, 28027 Madrid, Spain; adelafuente.1@unav.es

**Keywords:** cardiac magnetic resonance imaging, stress perfusion CMR, regadenoson, myocardial ischemia, late gadolinium enhancement, prognostic value

## Abstract

**Background/Objectives**: Regadenoson is increasingly used as a vasodilator stress agent for perfusion cardiac magnetic resonance (CMR) imaging due to its favorable pharmacologic profile. However, its long-term prognostic value in patients with myocardial ischemia remains insufficiently established. **Methods**: We retrospectively analyzed all regadenoson stress-CMR studies performed at our institution between May 2017 and July 2020, including patients with follow-up longer than three months. Inducible ischemia and late gadolinium enhancement (LGE) were assessed using standardized criteria. The primary composite endpoint included cardiovascular death, non-fatal myocardial infarction, late coronary revascularization (≥3 months after CMR), or hospitalization for unstable angina. Event-free survival was analyzed with Kaplan–Meier curves, and prognostic factors were evaluated using a Fine–Gray competing-risks model. **Results**: Of 705 examinations, 698 were eligible, and 517 patients (78.5%) completed follow-up over a median of 1.93 years (IQR 1.37–2.79). Inducible ischemia was identified in 142 patients (27.5%). During follow-up, 38 composite events occurred. Event incidence was significantly higher in patients with ischemia (109.6 events/1000 person-years; 95% CI 75.7–158.7) than in those without (13.3 events/1000 person-years; 95% CI 7.2–24.7; RR 8.25; 95% CI 4.01–16.98; *p* < 0.001). In multivariable analysis, inducible ischemia independently predicted adverse outcomes (HR 4.50; 95% CI 1.86–10.9; *p* = 0.001), whereas LGE was not independently associated (HR 1.28; 95% CI 0.46–3.57; *p* = 0.63). **Conclusions**: Regadenoson stress-CMR provides robust medium-term risk stratification in patients with suspected or known coronary artery disease. Detection of inducible ischemia strongly predicts major cardiovascular events, underscoring its prognostic and clinical relevance.

## 1. Introduction

Coronary artery disease (CAD) is one of the main health problems worldwide. It is estimated that more than 126 million individuals are affected globally (1.72% of the world’s population), accounting for approximately 9 million deaths per year and representing the leading cause of disability and years of life lost [1]. Early and accurate diagnosis enables timely initiation of appropriate therapy, thereby reducing morbidity and mortality [2].

Perfusion imaging with cardiac magnetic resonance (CMR) has proven high diagnostic performance, both for detecting angiographically significant coronary stenosis (≥50% luminal obstruction) [3,4,5] and for identifying hemodynamically relevant lesions as defined by fractional flow reserve (FFR) [6,7,8]. Comparative studies show that stress-CMR offers superior diagnostic accuracy to most non-invasive tests, with performance comparable only to PET [2,9,10,11,12,13].

Regadenoson is a selective A2A adenosine receptor agonist with vasodilatory efficacy comparable to adenosine [14,15,16] and superior to dipyridamole [17]. Because of its more specific pharmacologic profile [18,19], regadenoson is generally better tolerated [20,21] and constitutes a safer alternative in specific vulnerable populations, including patients with bronchial hyperreactivity [22,23,24], heart transplant recipients [25,26,27], and individuals at increased risk for atrioventricular block [15,20,21,28,29].

Vasodilator stress-CMR has shown high prognostic value in patients with known or suspected CAD, as demonstrated in the SPINS study [30]. Most available prognostic data derive from studies using dipyridamole [31,32] or adenosine [33,34,35], whereas evidence specifically addressing regadenoson stress-CMR remains limited [36,37].

The aim of this study was to evaluate the prognostic value of regadenoson stress perfusion CMR in an unselected clinical population.

## 2. Materials and Methods

### 2.1. Study Population

A total of 705 stress-CMR examinations performed between May 2017 and July 2020 were screened [28]. Hemodynamically unstable patients, those with myocardial infarction < 24 h before the examination, and those with contraindications to regadenoson or gadolinium injection (e.g., GFR < 30 mL/min/1.73 m^2^ or pregnancy) were excluded. Baseline characteristics and outcomes were extracted from electronic medical records.

All patients provided written informed consent. The study was approved by the institutional Drug Research Ethics Committee, in accordance with Royal Decree 957/2020 and the Declaration of Helsinki (CUN-REG-2019-01, Approval date: 4 February 2019). Due to the retrospective nature of the study, written informed consent was waived by the Institutional Review Board.

### 2.2. CMR Protocol

The CMR protocol followed validated institutional standards [28,38]. Patients abstained from methylxanthine-containing substances for 24 h before scanning [39,40,41]. Imaging was performed on a 1.5 T Magnetom Aera scanner (Siemens Healthineers, Erlangen, Germany) using a standard stress–rest perfusion protocol with cine steady-state free precession, first-pass perfusion, and late gadolinium enhancement. Regadenoson (Rapiscan, GE Healthcare AS) 0.4 mg was administered intravenously, and stress perfusion imaging commenced 70 s thereafter. Aminophylline 200 mg IV was given approximately 150 s after regadenoson to reverse vasodilator-induced hyperemia. Gadobutrol (Gadovist, Bayer AG, Berlin, Germany) was injected at 0.15 mmol/kg at 4 mL/s.

### 2.3. CMR Image Analysis

Image analysis was conducted using dedicated software (cvi42, Circle Cardiovascular Imaging Inc., Calgary, AB, Canada). Myocardial ischemia was defined as a subendocardial perfusion defect following a coronary artery distribution, with normal perfusion at rest and absence of late gadolinium enhancement (LGE). For LGE assessment, both presence and distribution were analyzed. LGE patterns were categorized as ischemic (subendocardial or transmural) or non-ischemic. Transmural LGE was defined as >50% myocardial wall thickness involvement. Analysis of myocardial ischemia and LGE was performed by two certified radiologists in consensus, blinded to clinical data.

### 2.4. Analysis of Events

Perfusion CMR findings (presence or absence of inducible ischemia) were used for the prognostic analysis. Adverse events included all-cause mortality, cardiovascular death, non-fatal myocardial infarction, coronary revascularization performed at least 3 months after CMR, hospitalization for unstable angina, hospitalization for heart failure, and ischemic stroke. The composite endpoint comprised cardiovascular death, non-fatal myocardial infarction, late coronary revascularization, and hospitalization for unstable angina. Because coronary revascularization was one of the outcome events, procedures performed within the first 3 months after stress-CMR were considered to be directly triggered by the test and were therefore not counted as follow-up events, in line with previous studies [32].

### 2.5. Statistical Analysis

Continuous variables were expressed as mean ± standard deviation and compared using the independent-samples *t* test, whereas categorical variables were compared using the chi-square test. The incidence of all events and their 95% confidence intervals was calculated in the overall cohort and separately according to the presence or absence of inducible ischemia on stress-CMR. Corresponding *p* values for between-group comparisons were reported, and the same analyses were repeated according to the presence or absence of LGE.

The prognostic value of stress-CMR was evaluated for the composite endpoint. Event-free survival in patients with and without inducible ischemia was estimated using Kaplan–Meier curves and compared with the log-rank test. Additionally, a competing-risks regression model was fitted according to the Fine–Gray approach, modeling the cumulative incidence of the composite endpoint while accounting for non-cardiovascular death as a competing event [42]. Inducible ischemia on stress-CMR was the main exposure and was examined alone and in combination with LGE status. In the multivariable model, all clinical variables associated with the outcome in univariable analysis (*p* < 0.10) were initially included as covariates and backwards procedure was used to optimize the model. Positive stress-CMR and presence of LGE were forced to remain in the model.

Sensitivity regression analyses with the same variables were done to evaluate the effect of follow-up losses. As there may be many possible best and worst scenarios depending on length of follow-up and competing events, these characteristics were fixed to simplify the analyses. Thus, all patients without follow-up were assigned the median follow-up time of the entire cohort. In the best-case scenario, none of these patients suffered the combined event, while, in the worst-case scenario, all patients suffered it at the end of follow-up. Furthermore, it was considered that none of the patients suffered the competing event in either scenario.

Deaths were classified into three categories, consistent with the methodology used in the SPINS study [30]: cardiovascular death, cancer-related death, and death from other or unknown causes. Cardiovascular death was defined as death preceded by acute myocardial infarction, malignant ventricular arrhythmias, or decompensated heart failure. Event adjudication was based on information obtained from electronic medical records.

All statistical analyses were performed using SPSS software (version 23.0, SPSS Inc., Chicago, IL, USA). A two-sided *p* value < 0.05 was considered statistically significant.

## 3. Results

### 3.1. Study Population

**Figure 1:**
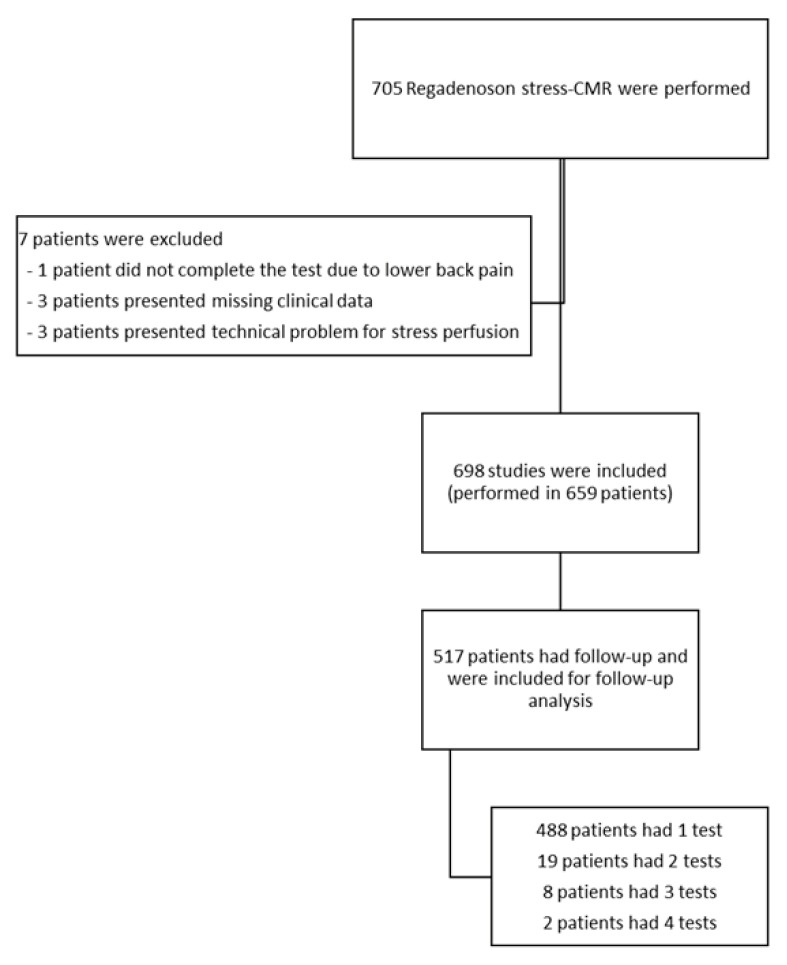
Flowchart of the study population.

Seven examinations were excluded from the analysis (four owing to incomplete stress-CMR studies and three due to missing clinical data), resulting in 698 evaluable stress-CMR scans in 659 patients. Follow-up data were available for 517 patients (78.5%), with a median follow-up of 1.93 years (interquartile range, 1.37–2.79 years). Most patients (n = 488) underwent a single examination, whereas 29 patients had repeated studies (Figure 1). Patients lost to follow-up had similar baseline characteristics, although those who completed follow-up were slightly older (clinical and demographic characteristics are shown in Table S1 of Supplementary Data).

### 3.2. Baseline Characteristics

Overall baseline characteristics and stratified by the presence or absence of inducible ischemia on stress-CMR are summarized in Table 1. Among the 517 patients with follow-up, 142 (27.5%) demonstrated inducible ischemia, with a mean of 4.3 ± 3.3 ischemic segments. Demographic and clinical variables were generally similar between patients with and without inducible ischemia. Patients with inducible ischemia were slightly older.

### 3.3. CMR Findings

**Figure 2:**
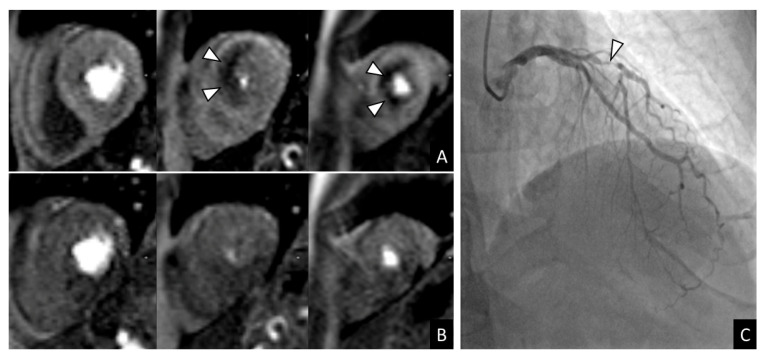
Stress-CMR in a 73-year-old man (former smoker with hypertension and diabetes) presenting with recurrent episodes of sustained and non-sustained ventricular tachycardia demonstrated an anteroseptal perfusion defect during stress ((**A**), arrowheads) that was not present at rest (**B**). Subsequent coronary angiography (**C**) revealed a severe mid–left anterior descending coronary artery stenosis, which was treated with implantation of a drug-eluting stent.

Baseline CMR characteristics stratified by the presence or absence of inducible ischemia are summarized in Table 2. Patients with inducible ischemia had similar left ventricular size and ejection fraction but a higher prevalence of ischemic LGE, higher right ventricular ejection fraction, and more frequent concentric hypertrophy and left ventricular dilatation. Representative perfusion and LGE images are shown in Figure 2.

### 3.4. Follow-Up

During a median follow-up of 1.93 years (interquartile range, 1.37–2.79 years), 38 composite events occurred (incidence rate of 37.7 events per 1000 person-years, 95% CI, 27.4–51.8). Event-specific incidence rates are summarized in Table 3.

### 3.5. Mortality

There were 26 deaths in total: 1 cardiovascular death, 14 cancer-related deaths, 1 ischemic stroke, 1 due to porto-spleno-mesenteric thrombosis, and 9 deaths of unknown cause. Thirteen of these patients had a negative stress-CMR for inducible ischemia; only one of them underwent coronary angiography, which showed no significant coronary disease. Only one patient with a positive stress CMR result underwent coronary angiography, and no significant coronary stenoses were identified. Cancer type was available in 11 patients, and in all cases the malignancy had a poor prognosis based on clinical characteristics or advanced stage (stage IV in eight cases).

### 3.6. Stress-CMR and Outcomes

**Figure 3:**
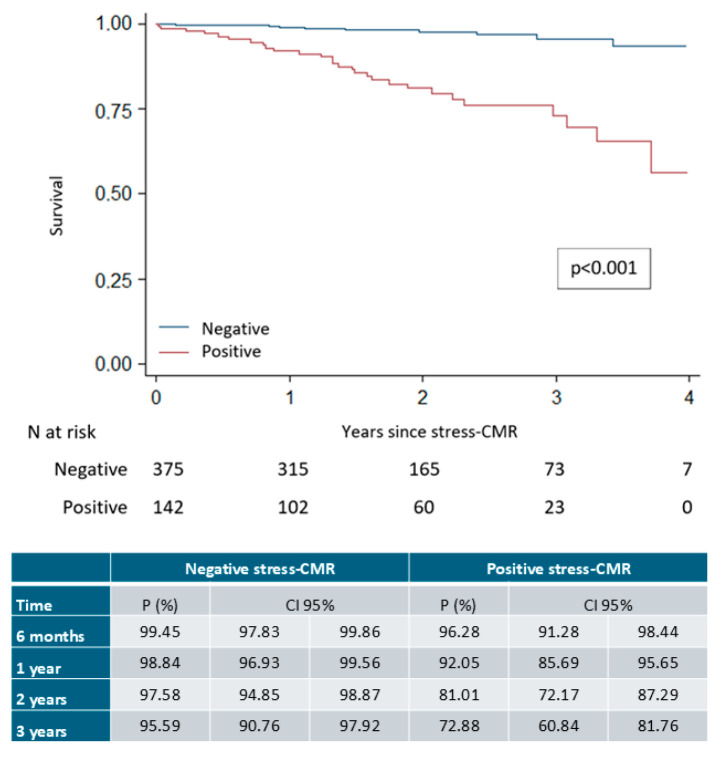
Survival curves for the composite event depending on the stress-CMR.

Inducible ischemia was strongly associated with the composite endpoint, with an incidence rate of 109.6 events per 1000 person-years (95% CI, 75.7–158.7), which was significantly higher than the rate in patients without inducible ischemia (13.3 events per 1000 person-years; 95% CI, 7.2–24.7; rate ratio 8.25; 95% CI, 4.01–16.98; *p* < 0.001). Event-specific incidence rates are summarized in Table 4, and Kaplan–Meier survival curves are shown in Figure 3. The table accompanying Figure 3 displays the proportion of event-free survival in both groups at 6 months and at 1, 2, and 3 years, with statistically significant differences seen from the first year onward and increasing over time (log-rank test, *p* < 0.001).

### 3.7. LGE and Outcomes

**Figure 4:**
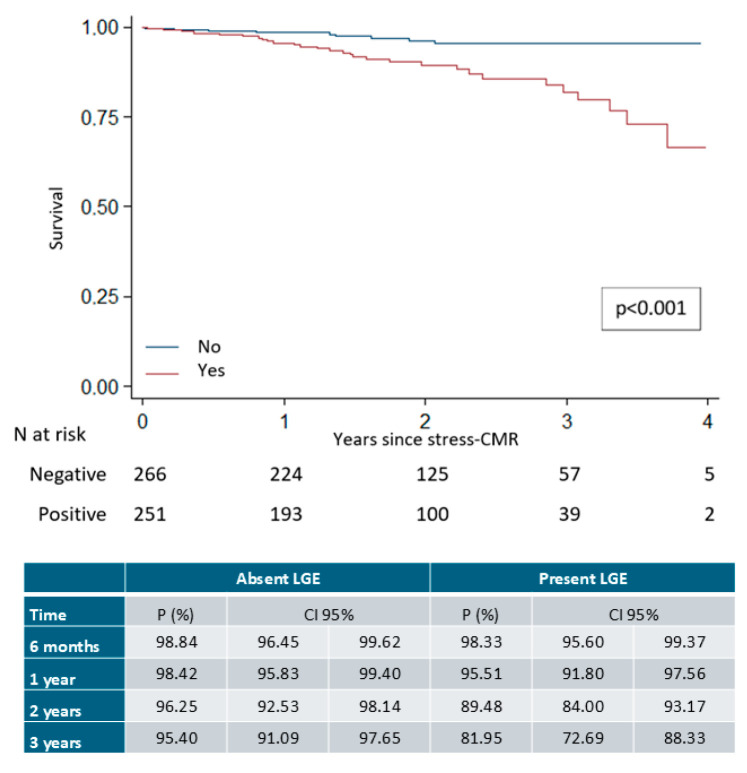
Survival curves for the composite event depending on the presence of late gadolinium enhancement.

LGE presence was associated with higher rates of the composite endpoint, with an incidence of 63.1 events per 1000 person-years (95% CI, 43.9–90.1), significantly higher than in patients without LGE (16.4 events per 1000 person-years; 95% CI, 8.5–31.5; rate ratio 3.8; 95% CI, 1.8–8.1; *p* < 0.001). Detailed incidence rates are presented in Table 5, and time-to-event curves are shown in Figure 4.

### 3.8. Regression Analysis

In univariate competing-risks model analysis, inducible ischemia, LGE, dyslipidemia, prior stent implantation, chronic kidney disease, and antiplatelet therapy were associated with the composite endpoint. In the multivariable model (Table 6), inducible ischemia remained independently associated with the composite event (HR 4.50; 95% CI, 1.86–10.9; *p* = 0.001), whereas the presence of LGE was not independently associated (HR 1.28; 95%, 95% CI, 0.46–3.57; *p* = 0.632).

## 4. Discussion

Stress-CMR provides several advantages as a first-line diagnostic modality in patients with suspected coronary artery disease, including comprehensive assessment of cardiac anatomy and function, detailed tissue characterization using parametric mapping and late gadolinium enhancement, and the absence of ionizing radiation [8,43,44,45,46]. Most of the available prognostic data come from studies using adenosine or dipyridamole as vasodilator agents [31,32,47], whereas regadenoson—despite its established vasodilatory efficacy and favorable tolerability profile—remains underrepresented in outcome-focused cohorts [36]. Large series such as SPINS did not report separate analyses for this agent [30], leaving its specific prognostic value insufficiently defined.

The present study analyzed a retrospective cohort of all regadenoson stress-CMR examinations performed at a single institution between 2017 and 2020, including 698 tests for baseline description and 517 patients (79.2%) with completed follow-up. Previous work from the same group has demonstrated the safety and feasibility of regadenoson in the setting of stress-CMR [28,38]. In this cohort, regadenoson stress-CMR enabled effective medium-term risk stratification, with patients exhibiting inducible ischemia showing a markedly higher incidence of adverse events and an approximately eight-fold increase in the composite event rate, highlighting the strong prognostic significance of stress-induced perfusion defects.

Total mortality was not included in the composite endpoint, although it was recorded and incorporated as a competing event in the regression model [42]. In line with the SPINS study [30], overall mortality was excluded from the composite outcome because of the expected low cardiac event rate and the characteristics of our study population. Most deaths occurred in patients without inducible ischemia, likely reflecting the institutional case mix, which includes a high proportion of oncology patients enrolled in clinical trials with potentially cardiotoxic therapies who may be referred for stress-CMR. Including total mortality in the composite endpoint, as in some dipyridamole—[32,48] and adenosine-based cohorts [33], could therefore have introduced bias into the analysis.

In this cohort, inducible ischemia, but not the presence of LGE, was independently associated with the composite endpoint. While LGE is widely recognized as a marker of irreversible myocardial injury and adverse prognosis, its relative prognostic contribution may vary according to the clinical context. Further, a binary LGE variable (present/absent) likely lacks sufficient granularity for risk stratification [49]. A recent study by Jones et al. [50] highlighted the importance of quantitative scar burden for predicting adverse outcomes.

Our study population had preserved left ventricular systolic function (mean LVEF 66%), in contrast to cohorts in which LGE robustly predicts arrhythmic or mortality outcomes [45,50,51,52]. Similarly, Lota et al. [53] found that non-ischemic LGE did not predict adverse events in individuals with preserved LVEF. This finding suggests that the ischemic burden be more directly linked to future adverse events in this population. Once inducible ischemia and global function are considered, the incremental value of LGE appears attenuated, which aligns with previous reports indicating that scar burden becomes dominant mainly in patients with severely reduced function or extensive infarction [54,55]. We acknowledge that the present analysis was also probably underpowered to detect LGE-related events such as arrhythmias or cardiovascular death, which were infrequent. Of note, no patient without inducible ischemia and without LGE experienced events, underscoring the high negative predictive value of a completely normal regadenoson stress-CMR examination [11]. Overall, these results highlight the primary prognostic importance of functional ischemia over permanent scar in the risk stratification of patients.

This retrospective study has limitations that are inherent to its design. Clinical data were obtained from electronic medical records that were not specifically designed for research, which may have led to missing information, particularly regarding medication adherence, risk factor control, and changes in therapy after stress-CMR. Because CMR findings likely influenced subsequent clinical management, event rates among higher-risk patients may have been attenuated. A positive stress-CMR result may prompt closer clinical surveillance and more intensive medical treatment, which could improve prognosis in these patients. Consequently, any bias related to incomplete information would be expected to underestimate, rather than exaggerate, differences between groups. Therefore, the true differences in outcomes may be larger than those observed. Follow-up was achieved in 79.2% of patients (median duration of 1.93, IQR, 1.37–2.79 years). The study was conducted at a private institution outside the public healthcare system, which may have limited the completeness of follow-up because it depended on patients returning to the center for scheduled visits. Baseline characteristics did not significantly differ between patients with and without follow-up, limiting the possibility of bias due to follow-up losses. In addition, sensitivity analyses done under unlikely extreme assumptions confirm the predictive value of positive stress-CMR, although they suggest that the strength of the association, if anything, may be somewhat overestimated. 

## 5. Conclusions

Regadenoson stress-CMR enabled effective mid-term risk stratification in an unselected cohort with suspected or known coronary artery disease. Inducible ischemia demonstrated a strong, independent association with adverse clinical outcomes, whereas the presence of LGE did not confer additional prognostic information beyond perfusion findings. These results support regadenoson stress-CMR as a robust modality for ischemia assessment and clinical decision-making in routine practice.

## Figures and Tables

**Table 1:** Baseline characteristics of the study population.

	Follow-Up (n = 517)	Ischemia Absent (n = 375)	Ischemia Present (n = 142)	*p*-Value
Demographics				
Age (years)	65.2 ± 11.2	64.3 ± 11.3	67.4 ± 10.7	0.007
Elderly (≥70 years) (%)	203 (39.3)	137 (36.5)	66 (46.5)	0.039
Gender (female/male) (%)	128 (24.8)	98 (26.1)	30 (21.1)	0.239
Height (m)	1.69 ± 0.08	1.7 ± 0.08	1.68 ± 0.08	0.123
Weight (kg)	78.5 ± 14.1	80.2 ± 15.2	74.3 ± 12.7	<0.001
BMI (kg/m^2^)	27.4 ± 4.5	27.8 ± 4.6	26.2 ± 3.8	<0.001
Body surface area (m^2^)	1.91 ± 0.21	1.93 ± 0.2	1.86 ± 0.19	<0.001
Sinus Rhythm (%)	73.1	258 (68.8)	120 (84.5)	<0.001
Cardiovascular risk factors				
Smoker/former smoker (%)	295 (57.1)	209 (55.7)	86 (60.6)	0.322
Hypertension (%)	315 (60.9)	221 (58.9)	94 (66.2)	0.131
Dyslipidemia (%)	319 (61.7)	218 (58.1)	101 (71.1)	0.007
Diabetes mellitus (%)	125 (24.2)	83 (22.1)	42 (29.6)	0.078
Obesity (BMI ≥30 Kg/m^2^) (%)	122 (23.6)	102 (27.2)	20 (14.1)	0.002
Family history of CAD (%)	136 (26.3)	92 (24.5)	44 (31.0)	0.137
Prior coronary bypass (%)	26 (5.0)	7 (1.9)	19 (13.4)	<0.001
Prior coronary stent (%)	133 (25.7)	72 (19.2)	61 (43.0)	<0.001
Chronic kidney disease (%)	25 (4.8)	16 (4.3)	9 (6.3)	0.327
COPD/Asthma (%)	68 (13.2)	52 (13.9)	16 (11.3)	0.435
OSAHS (%)	52 (10.1)	45 (12.0)	7 (5.0)	0.018
Medication				
ACEi/ARBs (%)	235 (45.5)	164 (43.7)	71 (50.0)	0.202
Aspirin (%)	217 (42.0)	126 (33.6)	91 (64.1)	<0.001
Antiplatelet P2Y12 (%)	74 (14.3)	36 (9.6)	38 (26.8)	<0.001
Oral anticoagulation (%)	83 (16.1)	66 (17.6)	17 (12.0)	0.120
Beta-blockers (%)	204 (39.5)	131 (34.9)	73 (51.4)	0.001
Clinical indication for stress-CMR				
Previous revascularization (%)	151 (29.2)	78 (20.8)	73 (51.4)	<0.001
Angina or equivalent (%)	110 (21.3)	89 (23.7)	21 (14.8)	
Suspected cardiomyopathy (%)	124 (24.0)	105 (28.0)	19 (13.4)	
High risk profile (%)	29 (5.6)	24 (6.4)	5 (3.5)	
Ventricular tachycardia (%)	30 (5.8)	26 (6.9)	4 (2.8)	
Previous CCTA or exercise ECG (%)	51 (10.0)	35 (9.3)	16 (11.3)	
Heart transplant (%)	22 (4.3)	18 (4.8)	4 (2.8)	

Note. Data are presented as mean ± standard deviation or as percentages (%). m: meter; kg: kilogram; BMI: body mass index; CAD: coronary artery disease; COPD: chronic obstructive pulmonary disease; OSAHS: obstructive sleep apnea/hypopnea syndrome; ACEi: angiotensin-converting enzyme inhibitors; ARBs: Angiotensin II receptor blockers; CMR: cardiac magnetic resonance; CCTA: coronary computed tomography angiography; ECG: electrocardiogram.

**Table 2:** CMR findings based on stress testing results.

	Ischemia Absent (n = 375)	Ischemia Present (n = 142)	*p*-Value
Morphology and function			
V EF, %	66.4 ± 12.4	64.9 ± 13.8	0.336
LV ESVI, mL/m^2^	25.8 ± 18.4	28.3 ± 20.8	0.381
LV EDVI, mL/m^2^	71.9 ± 22.9	73.3 ± 22.9	0.447
LV mass index, g/m^2^	69.7 ± 18.1	71.5 ± 14.9	0.085
RV EF, %	62.4 ± 9.0	65.0 ± 8.8	0.004
RV ESVI, mL/m^2^	25.7 ± 18.5	27.9 ± 20.3	0.41
RV EDVI, mL/m^2^	71.4 ± 19.2	69.3 ± 14.5	0.724
Left ventricular morphology			
Normal	55.5	47.9	<0.05
Concentric remodeling	17.9	11.3	
Asymmetric hypertrophy	4.3	5.6	
Concentric hypertrophy	9.3	18.3	
Eccentric hypertrophy	7.5	5.6	
Dilated	5.6	12.0	
Fibrosis—n (%)			
LGE ischemic pattern	16.5	54.9	<0.001
LGE non-ischemic pattern	24.3	14.1	0.012
Segments affected by LGE *, mean ± SD	3.5 ± 3.5	4.2 ± 2.8	0.002

Note. Data are presented as mean ± standard deviation or as percentages (%). CMR: cardiac magnetic resonance, LV: left ventricle, RV: right ventricle; ESVI = end systolic volume index, EDVI: end diastolic volume index; LGE = late gadolinium enhancement; EF: ejection fraction. * On tests with LGE.

**Table 3:** Global incidence rate of the different events. Rate expressed in 1000 person-years.

	GLOBAL (n = 517)		
	Person-Years	n	Incidence Rate (CI 95%)
Death	1051.33	26	24.7 (16.8–36.3)
CV death	1051.33	1	0.95 (0.13–6.8)
AMI	1047.7	5	4.8 (2–11.5)
Unstable angina	1044.2	4	3.83 (1.4–10.2)
Revascularization	1011.9	34	33.6 (24–47)
Composite event	1008	38	37.7 (27.4–51.8)
HF hospitalization	1036.5	14	13.5 (8–22.8)
Cerebrovascular event	1049.33	2	1.91 (0.48–7.6)

Note. CV: cardiovascular; AMI: acute myocardial infarction; HF: heart failure.

**Table 4:** Incidence rate of the different events based on the stress MRI perfusion result. Rate expressed in 1000 person-years.

	Negative Stress-CMR (n = 375)			Positive Stress-CMR (n = 142)			RR (CI 95%)	*p*-Value
	Person-Years	n	Incidence Rate (CI 95%)	Person-Years	n	Incidence Rate (CI 95%)		
Death	767.4	21	27.4 (17.8–42.0)	283.9	5	17.6 (7.3–42.3)	0.64 (0.2–1.7)	0.372
AMI	767.3	1	1.3 (0.2–9.3)	280.4	4	14.3 (5.4–38.0)	11 (1.2–98)	0.007
Unstable angina	766.5	1	1.3 (0.2–9.3)	277.7	3	10.8 (3.5–33.5)	8.3 (0.86–79.1)	0.028
Revascularization	753.5	9	11.9 (6.2–23)	258.4	25	96.8 (65.3–143)	8.1 (3.8–17.4)	<0.001
Composite event	752.5	10	13.3 (7.2–24.7)	255.4	28	109.6 (75.7–158.8)	8.3 (4–17)	<0.001
HF hospitalization	756.2	9	11.9 (6.2–22.9)	280.3	5	17.8 (7.4–42.9)	1.5 (0.5–4.5)	0.465

Note. For cardiovascular death and stroke, the incidence in the different subgroups was not calculated due to the small number of events. CMR: cardiac magnetic resonance; AMI: acute myocardial infarction; HF: heart failure.

**Table 5:** Incidence rate of the different events based on the late gadolinium enhancement result. Rate expressed in 1000 person-years.

	Absent L266.			Present LGE (n = 251)			RR (CI 95%)	*p*-Value
	Person-Years	n	Incidence Rate (CI 95%)	Person-Years	n	Incidence Rate (CI 95%)		
Death	557.1	13	23.3 (13.6–40.2)	494.3	13	26.3 (15.3–45.3)	1.1 (0.52–2.4)	0.760
AMI	557.1	0	0	490.6	5	10.2 (4.2–24.5)		
Unstable angina	557.1	0	0	487.1	4	8.2 (3.1–21.9)		
Revascularization	548.7	9	16.4 (8.5–31.5)	463.2	25	54 (36.5–79.9)	3.3 (1.5–7.1)	0.001
Composite event	548.7	9	16.4 (8.5–31.5)	459.3	29	63.1 (43.9–90.1)	3.8 (1,8–8.1)	<0.001
HF hospitalization	550.7	7	12.7 (6.1–26.7)	485.8	7	14.4 (6.9–30.2)	1.1 (0.4–3.2)	0.815

Note. For cardiovascular death and stroke, the incidence in the different subgroups is not calculated due to the small number of events. AMI: acute myocardial infarction; HF: heart failure.

**Table 6:** Initial and final multivariate models for the composite event using a competing risks model.

	Initial Model			Final Model		
	**sHR**	**95% C.I.**	***p*** **-Value**	**sHR**	**95% C.I.**	***p*** **-Value**
Positive stress-CMR	4.50	1.86–10.9	0.001	4.88	1.91–12.5	0.001
Presence of LGE	1.28	0.46–3.57	0.632	1.34	0.48–3.77	0.573
Sinus Rhythm *	2.19	0.67–7.15	0.193			
Dyslipidemia	1.29	0.58–2.91	0.532			
Diabetes mellitus	1.32	0.63–2.78	0.464			
Prior coronary stent	0.69	0.29–1.62	0.395			
Chronic kidney disease	2.78	0.94–8.20	0.064	3.20	1.23–8.34	0.018
Aspirin	3.25	1.31–8.10	0.011	3.0	1.30–6.92	0.010
Antiplatelet P2Y12	3.12	1.50–6.51	0.002	2.85	1.37–5.97	0.005
Beta-blockers	1.32	0.61–2.87	0.478			

Note. CMR: cardiac magnetic resonance; LGE: late gadolinium enhancement; sHR: subhazard ratio; C.I.: confidence interval. * Sinus rhythm = absence of conduction disorders or rhythm disorders.

## Data Availability

The datasets presented in this article are not readily available because of privacy or ethical restrictions. Requests to access the datasets should be directed to Gorka Bastarrika.

## Supplementary Materials

The following supporting information can be downloaded at: https://www.mdpi.com/article/10.3390/medsci14020190/s1, Table S1. Clinical and demographic characteristics of the study cohort according to follow-up status.

## Abbreviations

The following abbreviations are used in this manuscript:

CAD	coronary artery disease
CMR	cardiac magnetic resonance
FFR	fractional flow reserve
PET	positron emission tomography
GFR	glomerular filtration rate
LGE	late gadolinium enhancement
LV	left ventricle/left ventricular
LVEF	left ventricular ejection fraction
RV	right ventricle/right ventricular
SPINS	Stress-CMR Perfusion Imaging in the United States (multicenter registry/study name)
IV	intravenous
SD	standard deviation
CI	confidence interval
