# Supplementary material for: Prognostic Value of Regadenoson Stress Perfusion CMR
Source: Med Sci (Basel). 2026 Apr 10;14(2):190. doi: 10.3390/medsci14020190 (PMC13108122; doi:10.3390/medsci14020190)
Supplement: Supplementary file 1 [file medsci-14-00190-s001.zip › medsci-4179261-supplementary.pdf]

SUPPLEMENTARY DATA

Table S1. Clinical and demographic characteristics of the study cohort according to follow-up status.

Profile	No follow-up (n=136)	Follow-up (n=517)	p value
Age (years)	61.9 ± 12.6	65.2 ± 11.2	0.002
Older (≥70 years) (%)	39 (28.7)	203 (39.3)	0.023
Sex (female/male) (%)	34 (25.0)	128 (24.8)	0.954
Height (m)	1.69 ± 0.1	1.69 ± 0.08	0.831
Weight (kg)	79.7 ± 15.4	78.5 ± 14.1	0.461
BMI (kg/m²)	27.9 ± 4.7	27.4 ± 4.5	0.278
Body surface area (m²)	1.93 ± 0.22	1.91 ± 0.21	0.507
Sinus rhythm (%)	78.7	73.1	0.187
**Cardiovascular risk factors**			
Smoking (%)	76 (55.9)	295 (57.1)	0.805
Hypertension (%)	79 (58.1)	315 (60.9)	0.547
Dyslipidemia (%)	86 (63.2)	319 (61.7)	0.743
Diabetes mellitus (%)	33 (24.3)	125 (24.2)	0.983
Obesity (BMI ≥30 kg/m²) (%)	39 (28.7)	122 (23.6)	0.221
Family history of CAD (%)	48 (35.3)	136 (26.3)	0.038
Chronic kidney disease (%)	4 (2.9)	25 (4.8)	0.340
COPD/Asthma (%)	16 (11.8)	68 (13.2)	0.667
OSAHS (%)	10 (7.4)	52 (10.1)	0.332
**Previous treatment**			
Previous coronary bypass (%)	4 (2.9)	26 (5.0)	0.301
Previous coronary stent (%)	37 (27.2)	133 (25.7)	0.726
**Medication**			
ACE inhibitors/ARBs (%)	66 (48.5)	235 (45.5)	0.522
Aspirin (%)	66 (48.5)	217 (42.0)	0.170
P2Y12 inhibitor antiplatelets (%)	27 (19.9)	74 (14.3)	0.112
Oral anticoagulation (%)	15 (11.0)	83 (16.1)	0.144
Beta-blockers (%)	52 (38.2)	204 (39.5)	0.795
**Indication for stress-CMR – n (%)**			0.021
Previous revascularization	45 (33.1)	151 (29.2)	
Angina or angina equivalent	34 (25.0)	110 (21.3)	
Suspected cardiomyopathy	37 (27.2)	124 (24.0)	
High cardiovascular risk	9 (6.6)	29 (5.6)	
Ventricular tachycardia	6 (4.4)	30 (5.8)	
Previous exercise test or CCTA	5 (3.7)	51 (10.0)	
Heart transplantation	0	22 (4.26)	

Note: Data are presented as mean ± standard deviation or percentages. m: meter; kg: kilogram; BMI: body mass index; CAD: coronary artery disease; COPD: chronic obstructive pulmonary disease; OSAHS: obstructive sleep apnea-hypopnea syndrome; ACEI: angiotensin-converting enzyme inhibitor; ARB: angiotensin II receptor blocker.
